# Involvement of the intrinsic functional network of the red nucleus in complex behavioral processing

**DOI:** 10.1093/texcom/tgac037

**Published:** 2022-08-25

**Authors:** Yul-Wan Sung, Sachiko Kiyama, Uk-Su Choi, Seiji Ogawa

**Affiliations:** Kansei Fukushi Research Institute, Tohoku Fukushi University, Sendai, Miyagi 9893201, Japan; Department of Linguistics, Tohoku University, Sendai, Miyagi 9800862, Japan; Medical Device Development Center, Daegu-Gyeongbuk Medical Innovation Foundation, Daegu 41061, Republic of Korea; Kansei Fukushi Research Institute, Tohoku Fukushi University, Sendai, Miyagi 9893201, Japan

**Keywords:** functional network, musical training, interpersonal reactivity index, resting state functional MRI, red nucleus

## Abstract

Previous studies suggested the possibility that the red nucleus (RN) is involved in other cognitive functions than motion per se, even though such functions have yet to be clarified. We investigated the activation of RN during several tasks and its intrinsic functional network associated with social cognition and musical practice. The tasks included finger tapping, n-back, and memory recall tasks. Region of interest for RN was identified through those tasks, anatomical information of RN, and a brain atlas. The intrinsic functional network was identified for RN by an analysis of connectivity between RN and other regions typically involved in seven known resting state functional networks with RN used as the seed region. Association of the RN network with a psychological trait of the interpersonal reactivity index and musical training years revealed subnetworks that included empathy related regions or music practice related regions. These social or highly coordinated motor activity represent the most complex functions ever known to involve the RN, adding further evidence for the multifunctional roles of RN. These discoveries may lead to a new direction of investigations to clarify probable novel roles for RN in high-level human behavior.

## Introduction

The red nucleus (RN) in human is thought to play less important roles than in vertebrates, where RN is involved in controlling gait and other forms of motion (Bunday et al. 2014). However, even in humans, RN retained limited control of the motion of body parts, such as hand motion, although this does not include the fine control of the fingers. The involvement of RN in motion control is also predicted by the existence of projection pathway, that involves the majority of RN projection axons, that relays information from the motor cortex to the cerebellum through the inferior olivary complex (Tokuno et al. 1995; Bunday et al. 2014; Cacciola et al. 2019). Furthermore, functional MRI (fMRI) studies have yielded evidence for the role of RN in motion control, especially in rather complicated motions. RN was shown to be activated by a finger tapping task, with RN activity modulated by pacing of the tapping speed and by the direction of movement (Silva et al. 2018). RN was also shown to be activated by oral movements as well as during grasping motion (Sörös et al. 2006; Habas and Cabanis 2007), and even by swallowing and breathing (Ackland et al. 1997; Satoh et al. 2015).

In addition to motion, RN is also activated by cognitive tasks, such as discrimination, as well as synchronization tasks (Liu et al. 2000; Cunningham and Zelazo 2007; Habas and Cabanis 2007; Xu et al. 2016). The possibility that RN was involved in cognitive processing was also deduced from anatomical studies. Further support was obtained from a lesion study showing that RN infarction resulted in various cognitive deficits, such as intellectual fatigability, decreased verbal fluency, and discrete memory impairment (Lefebvre et al. 1993; Miller et al. 1993; Lavezzi et al. 2009).

Although these previous studies suggested the possibility that RN was involved in high cognitive function, this is yet to be clarified through the use of specific psychological measures. In this study, we aimed to investigate RN function through intrinsic functional networks and psychological measures as well as several tasks. We examined the intrinsic functional network of RN as reflected in the resting state fMRI (rsfMRI). Of the various known intrinsic functional networks, we selected the default mode, salience, visual, sensorimotor, language, dorsal-attention, frontoparietal, and cerebellum networks for targeting specific candidate regions and examined their functional connectivity with RN for the identification of an intrinsic RN network. We examined the resulting network in association with a psychological trait of the interpersonal reactivity index (IRI) and musical training years, inspired by the findings in previous studies that RN was activated by intentional face misidentification and also reflects emotional maturation (Bhatt et al. 2009; Tamura et al. 2012), and that RN was activated by sound separation and was also connected with the auditory cortex (Alain et al. 2005; Lovell et al. 2014).

## Methods

The study was designed for task fMRI and resting state fMRI experiments. Task fMRI experiments were conducted for the finger tapping, n-back, and memory recall tasks. Two resting state experiments were conducted, one for subjects with IRI (Davis, 1980, 1983) and the other for subjects having musical practice experience. MRI scanner used in the experiments was a 3-Tesla Verio/Skyrafit scanner (Siemens, Erlangen, Germany). All participants provided written informed consent. All experiments were approved by the Institutional Review Board of Tohoku Fukushi University. We have examined the brain mechanism of face and scene processing by fMRI, and we used face and scene as stimulation for n-bask and memory recall tasks. An exploratory approach was used in this study and separate fMRI experiments were made. Each task or resting state fMRI experiment was performed with different subject group from the others.

### Task fMRI experiments

#### Finger tapping task

Fifteen healthy volunteers (6 male, 9 female, ages 21.0 ± 2.6 years; all right-handed) participated. Finger tapping was performed by touching the thumb with the other four fingers. Taping by the left and right hands were interleaved during the scan. An experimental scan consisted of four cycles with 30-s stimulus blocks (left, right, left and right), in the way of a rest block following stimulus block preceded by a rest block at the start of the scan. Total scan time was 270-s. fMRI data were acquired using the following parameters: repetition time = 2000 ms, matrix size = 64 × 64, in-plane resolution = 3.4 × 3.4 mm^2^, slice thickness = 3.4 mm.

#### N-back task

Thirteen healthy volunteers (7 males and 6 females, ages 24.0 ± 3.8 years) participated. N-back task consisted of three load levels (0-, 1-, and 2-back). A button was pressed for every matching condition. fMRI data were acquired using the following parameters: repetition time = 2000 ms, matrix size = 64 × 64, in-plane resolution = 3.4 × 3.4 mm^2^, slice thickness = 3.4 mm. Facial images generated by a software FaceGen (Singular Inversions, Toronto, Canada) were used as visual stimuli for the matching task. A block design paradigm was used with 12-s stimulation blocks and 18-s control (rest) blocks, with the stimulation block repeated three times. Total scan time was 120-s, including a 12-s dummy block, three stimulus blocks, and four rest blocks. Each stimulation block consisted of eight images. On duration of each image was 50 ms. The scans were conducted separately for the three (0-, 1-, and 2-back) tasks, with scan order randomized between the subjects.

#### Memory recall task

We reanalyzed fMRI data collected in a previous study (Sung et al. 2007). A total of 26 healthy volunteers (13 males, 13 females, ages 22.4 ± 2.2 years) participated. The 122-s experimental run comprised an initial resting state of 30-s, two 15-s trials with an inter-trial interval of 31-s, and a post-stimulus resting state of 31-s. The start of each trial was announced by one brief sound burst played through headphones, and the end of each trial, by two brief sound bursts. The duration of the 3.5 kHz bursts was 8 ms; the two end bursts were separated by 1000 ms in time. Subjects started to recall faces or buildings at the first signal and stopped at the second signal. Subjects closed their eyes through the experimental run. Two runs were performed for each subject.

### Resting state fMRI experiments

For the RN network, we calculated the correlation of the 32 brain regions (Table 1) involved in the DMN, salience, visual, sensorimotor, language, dorsal-attention, frontoparietal, and cerebellum networks, with RN as a seed. We used this approach because those networks are used as template networks in CONN, a toolbox for fMRI data processing, and functions reflected by those networks are considered to be related to IRI and music practicing.

**Table 1:** List of the 32 brain regions included in the networks of CONN (NMI coordinates)

**1**	**DefaultMode.MPFC (1,55,-3)**
**2**	**DefaultMode.LP (L) (−39,-77,33)**
**3**	**DefaultMode.LP (R) (47,-67,29)**
**4**	**DefaultMode.PCC (1,-61,38)**
**5**	**SensoriMotor.Lateral (L) (−55,-12,29)**
**6**	**SensoriMotor.Lateral (R) (56,-10,29)**
**7**	**SensoriMotor.Superior (0,-31,67)**
**8**	**Visual.Medial (2,-79,12)**
**9**	**Visual.Occipital (0,-93,-4)**
**10**	**Visual.Lateral (L) (−37,-79,10)**
**11**	**Visual.Lateral (R) (38,-72,13)**
**12**	**Salience.ACC (0,22,35)**
**13**	**Salience.AInsula (L) (−44,13,1)**
**14**	**Salience.AInsula (R) (47,14,0)**
**15**	**Salience.RPFC (L) (−32,45,27)**
**16**	**Salience.RPFC (R) (32,46,27)**
**17**	**Salience.SMG (L) (−60,-39,31)**
**18**	**Salience.SMG (R) (62,-35,32)**
**19**	**DorsalAttention.FEF (L) (−27,-9,64)**
**20**	**DorsalAttention.FEF (R) (30,-6,64)**
**21**	**DorsalAttention.IPS (L) (−39,-43,52)**
**22**	**DorsalAttention.IPS (R) (39,-42,54)**
**23**	**FrontoParietal.LPFC (L) (−43,33,28)**
**24**	**FrontoParietal.PPC (L) (−46,-58,49)**
**25**	**FrontoParietal.LPFC (R) (41,38,30)**
**26**	**FrontoParietal.PPC (R) (52,-52,45)**
**27**	**Language.IFG (L) (−51,26,2)**
**28**	**Language.IFG (R) (54,28,1)**
**29**	**Language.pSTG (L) (−57,-47,15)**
**30**	**Language.pSTG (R) (59,-42,13)**
**31**	**Cerebellar.Anterior (0,-63,-30)**
**32**	**Cerebellar.Posterior (0,-79,-32)**

#### Resting state experiment with IRI: RS_A

Participants consisted of 23 healthy volunteers for whom IRI was measured. All were college students registered in the same college (12 males and 11 females, ages 21.9 ± 2.0). For all subjects, rsfMRI data were acquired using the following parameters: repetition time = 1000 ms, matrix size = 64 × 64, in-plane resolution = 3.4 × 3.4 mm^2^, slice thickness = 3.4 mm, and number of volumes = 480. In the rsfMRI session, subjects were asked to lie on the bed, not to wander their mind, keep their eyes open, and gently focus their eyes on the center of the visual field. The lights in the room were turned off during MRI scan.

#### Resting state experiment with musical practice experience: RS_B

Twenty-one participants (3 males and 18 females, ages 20.0 ± 0.9) with musical training participated in this experiment. All were college students registered in the same college. Training period of participants ranged from 6 to 18 years in various musical instruments (piano, wind instruments, and percussion instruments). The imaging parameters were the same as in RS_A.

Structural images were acquired for each task or resting state fMRI experiment using the following parameters: repetition time = 1900 ms, echo time = 2.52 ms, matrix size = 256 × 256, in-plane resolution = 1 × 1 mm^2^, slice thickness = 1 mm, and number of slices = 192. Imaging was in the sagittal plane.

### Data analysis

Task fMRI data were analyzed by BrainVoyager QX (Brain Innovation B.V., Maastricht, the Netherlands) and the location of ROI was identified for RN. Correlation was calculated in Matlab (Mathworks Co, Natick, USA) between RN and the 32 ROI regions of the typical rsfMRI networks. The rsfMRI data were processed by DPABI and time courses were extracted from the ROIs using DPABI (Yan et al. 2016).

The image data of task fMRI were processed using the BrainVoyager QX (Brain Innovation BV, Postbus, the Netherlands) software. For all image data in the functional session for each subject motion correction was made by the 3D trilinear spline interpolation method (maximum threshold of 2 mm, 2 degrees) and slice scan time correction was made by the cubic spline method. Twodimensional images in the functional session were incorporated into 3D anatomical images through trilinear interpolation and transformed into Talairach space. For multi-subject analysis 3D Gaussian spatial smoothing (full width at half maximum (FWHM), 5.0 mm), linear trend removal and Gaussian temporal smoothing (FWHM, 2.8-s) were applied to the data sets. Multi-subject analysis was performed with a multi-subject random effect approach. Statistical analysis was performed with the procedure based on the general linear model (GLM) using the BrainVoyager. Each experimental condition (except for control) was defined as a separate predictor.

Resting state fMRI image data were preprocessed by resting-state fMRI software (DPABI) (Chao-Gan and Yu-Feng 2010; Yan et al. 2016). The pre-processing included slice-scan time correction, 3D motion correction (the maximum threshold of 1.5 mm and 1.5 degrees), removal of head motion effects by the Friston 24-parameter model, bandpass temporal filtering (between 0.01 and 0.1 Hz), and artifact rejection based on the CSF signals. Two-dimensional images in the functional session were incorporated into 3D anatomical images and spatially smoothed (full width at half maximum (FWHM), 5.0 mm). Correlation was calculated in Matlab (Mathworks Co, Natick, USA) between RN and the 32 ROI regions of the typical rsfMRI networks.

## Results

**Fig. 1:**
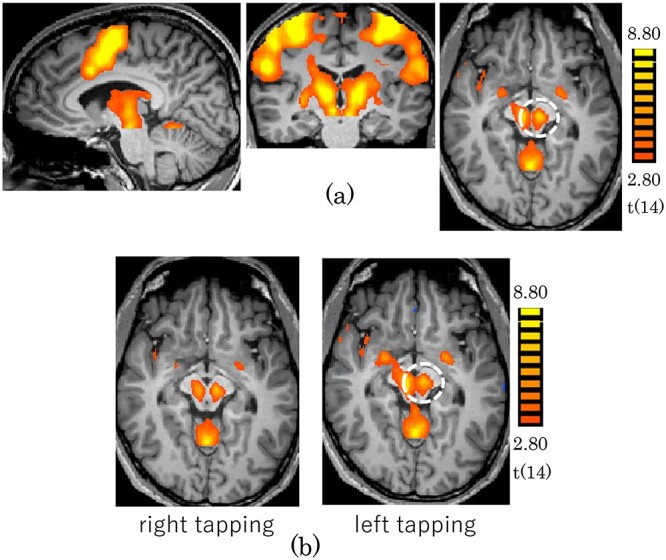
Activation maps obtained for the finger tapping task (*P* < 0.05; FDR correction). The dashed line encircles the ROI of RN with center coordinates (−8, −19, −8; MNI). (a) Tapping by the left and right hand (b) Tapping by the right hand (left) and the left hand (right).

**Fig. 2:**
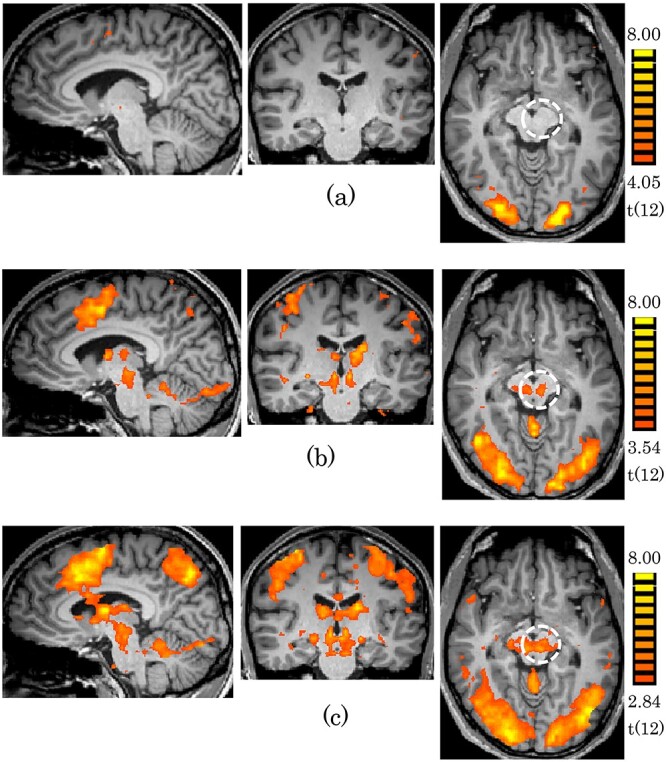
Activation maps obtained for the n-back tasks (*P* < 0.05; FDR correction). The dashed line encircles the ROI of RN with the center coordinates (−8, −19, −8; MNI). **(a)** 0-back (**b**) 1-back (**c**) 2-back.

**Fig. 3:**
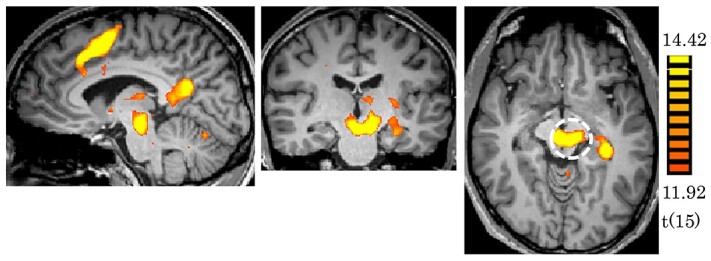
Activation maps by memory recall (*P* < 0.05; FDR correction). The dashed line encircles the ROI of RN with the center coordinates (−8, −19, −8; MNI).

The motor task experiments revealed activation at the midbrain bilaterally by finger tapping with both left and right hands. The activations by the left and right hands were similar (Fig. 1). The activation areas were closely matched with the anatomical location of RN (Supplementary Fig. 1). N-back task experiments revealed activation at the midbrain by 1-back and 2-back, but not by 0-back. The activation tended to be stronger for 2-back than for 1-back (Fig. 2). The memory recall task experiment revealed strong activation at the midbrain without external stimulation (Fig. 3). The location of the maps of the midbrain by N-back and memory recall tasks were almost the same as the one by the finger tapping task (Supplementary Fig. 1). The center of the activation map of the finger tapping task was at the coordinates of (−8, −19, −8; MNI). The location of the coordinates was proved as RN based on the Automated Anatomical Labeling (AAL3) (Supplementary Fig. 2).

**Fig. 4:**
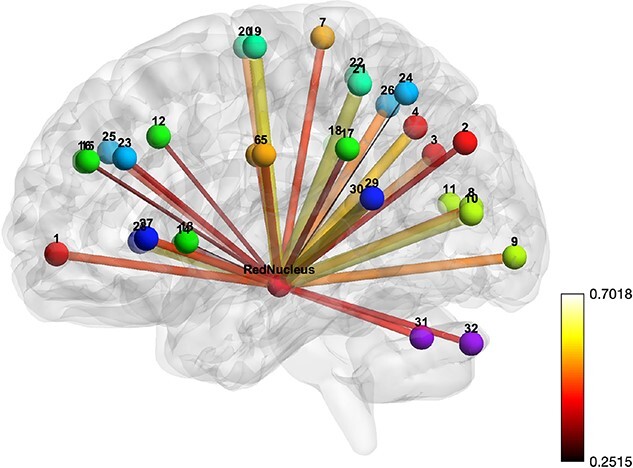
RN network. The bars represent the association of the edges with an IRI and the strength of association is represented by the thickness and color of the bar. Balls represent the centers of brain regions, each labeled by a distinct color. The color bar stands for correlation value.

**Fig. 5:**
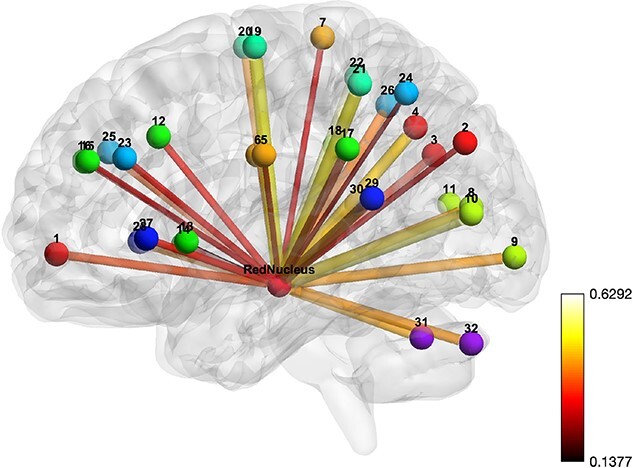
RN network. The bars represent the association of the edges with musical training years. The strength of association for an edge is represented by the thickness and color of the bar. Balls represent the centers of brain regions labeled by color. The color bar stands for correlation value.

A resting state network was derived by correlation of RN as the seed ROI with the 32 ROIs included in the typical rsfMRI networks (Table 1). The left RN was used as the seed because the activation of the left RN tended to be strong in all three fMRI experiments. The ROI for RN, to be used in the correlation analysis, was selected as a sphere of 5 mm diameter with center coordinates of (−8, −19, −8; MNI). Correlation of 32 ROIs with RN (32 edges) was calculated for each subject, and each edge was averaged over subjects separately for RS_A and RS_B. The mean correlation values of the 32 edges were significant for both RS_A and RS_B, resulting in two significant RN networks, each of 32 edges (*P* < 0.005, one sample t-test), one for RS_A (Fig. 4) and one for RS_B (Fig. 5), which proved the RN network consisted of 32 edges linking RN to all 32 regions.

Consistency of the RN network was confirmed by the strong and significant correlation (*r* = 0.93, *P* < 0.0001, corrected by permutation) between the networks obtained for RS_A and RS_B. For further evaluation of the consistency of the RN network, we used rsfMRI data acquired from 120 subjects who did not have their psychological trait index measured or musical experience (supplementary Method) to construct a third network with 32 edges. The correlation of the 32 edges of the third network with those of the first two (RS_A and RS_B) networks was very strong and significant (*r* = 0.98 and *r* = 0.96, respectively; *P* < 0.0001, corrected by permutation).

The association of the network with the psychological trait IRI (Supplementary Table 1) was significant at network edges 4 (DefaultMode.PCC) (*r* = −0.45; *P* = 0.03, corrected by permutation); 8 (Visual.Medial) (*r* = −0.48; *P* = 0.02, corrected by permutation); 12 (Salience.ACC) (*r* = −0.48; *P* = 0.03, corrected by permutation); 31 (Cerebellar.Anterior) (*r* = −0.46; *P* = 0.03); and 32 (Cerebellar.Posterior) (*r* = −0.48; *P* = 0.02, corrected by permutation).

A similar analysis revealed significant association of the network with the duration of musical training (Supplementary Table 2) at network edges 5 (SensoriMotor.Lateral (L)) (*r* = −0.46; *P* = 0.04, corrected by permutation); 6 (SensoriMotor.Lateral (R)) (*r* = −0.54; *P* = 0.02, corrected by permutation); and 28 (Language.IFG (R)) (*r* = −0.47; *P* = 0.04, corrected by permutation).

## Discussion

RN has been suggested to be involved in complicated motions and also various cognitive functions in human. Our observations made by task fMRI are consistent with previous reports related to motion and cognitive functions. The intrinsic functional network of RN identified in this study reflects interpersonal reactivity and periods of musical training experience. These results suggest that RN is involved in even high-level social behavior and highly coordinated complex motor behavior.

Activations by three tasks, finger tapping, n-back, and memory recall, at RN observed in our present study are consistent with previous studies. Our finger tapping task was not associated with any other tasks such as pacing in a reference rhythm or following direction, so our result suggests that RN is involved in this kind of motion control (Silva et al. 2018). Activation at RN by N-back task is likely to reflect working memory capacity (Kragel et al. 2019). In addition, the task was designed with face pictures, and brain regions for face processing might be involved in modulating the activation (Bhatt et al. 2009). Superior temporal sulcus is known to process face expression or related emotion (Narumoto et al. 2001). The modulation of activity by the load of task indicates that RN may play a role in association with other brain areas for processing such face information and memory related brain areas (Bhatt et al. 2009; Kragel et al. 2019). Activation by the memory recall task would be related to brain areas involved in processing faces and the buildings because the images participants tried to retrieve were faces or buildings. A previous study showed that brain areas specialized for faces or buildings were recruited while subjects were trying to recall a face or building (Sung and Ogawa 2008). Therefore, the activation at RN by the memory recall task suggests that RN may be connected with various brain areas of visual object processing and memory. These results together support the notion that RN is involved in cognitive functions as well as motor functions and has connections with brain areas responsible for those functions.

The resting state fMRI signal is known to be originated from coherent neuronal activities of the brain in the absence of stimulus tasks and thus reflects the intrinsic function of the brain (Biswal et al. 1995). The resting state functional network (connectivity) is defined by different brain regions with coherently fluctuating rsfMRI signals, and their connection is characterized by the strength of correlation. Resting state functional networks are known to reflect not only brain disorders such as Alzheimer’s disease, autism spectrum disorders, and schizophrenia but also cognitive functions related to social behaviors (Rachle et al. 2001; Muller et al. 2011; Agosta et al. 2012; Liang et al. 2012; Venkataraman et al. 2012; Li et al. 2013; Washington et al. 2014; Vigneshwaran et al. 2017). The significant correlation between RN and brain regions featuring in the DMN, salience, sensorymotor, visual, language, and cerebellum networks suggest a possibility of involvement of RN in brain functions that those networks are involved in.

The IRI is a measurement tool for the multi-dimensional assessment of empathy in relationships formed with others (Davis 1983). The subscales include perspective taking, empathic concern, fantasy, and personal distress. For brain regions in the RN network, previous studies support that those are related to IRI. The relation of the anterior cingulate cortex (ACC) to IRI is supported by previous anatomical and functional studies. ACC is known as one of the core brain regions responsible for empathy function (Jackson et al. 2006; Singer and Lamm 2009; Horan et al. 2016; Tomova et al. 2017). A previous study of brain anatomy reported that IRI was related to gray matter volume changes in ACC (Banissy et al. 2012). Studies that specifically investigated empathy for others’ pain showed that ACC activation could be modulated by the perception of other people (Mathur et al. 2016) and ACC was related to people’s ability to empathize with others (Lockwood et al. 2015). The relation of another brain region of the RN network, the cerebellum, to IRI is also supported by functional characters of the cerebellum involved in high-level cognitive functions, such as affection, emotion, and language, as well as motor functions (Leiner et al. 1993; Allen and Courchesne 1998; Schmahmann and Sherman 1998) in addition to an anatomical study in which IRI was associated with changes in volume of brain morphometry and mean diffusivity in the cerebellum (Picerni et al. 2021). The visual region of RN network is unexpectedly associated with IRI, but this kind of relatively primary region also reflects brain disorders, as identified in a study reporting hyperactivity of visual cortex in the resting state in patients with post-trauma syndrome disease (Zhu et al. 2014). Regarding the relation of the posterior cingulate cortex (PCC) in the RN network, no previous study has tied it directly with IRI. However, PCC has been known to be activated by emotional stimuli and firmly associated with emotional salience (Maddock et al. 2003; Brewer et al. 2013). A study of autism spectrum disorders demonstrated reduced functional coupling between the PCC and the medial prefrontal cortex, which raises the possibility that PCC is associated with IRI indirectly through ACC (Leung and Lau 2020).

The above results, together, raise the possibility that the subset of the RN network associated with IRI would be involved in higher cognitive functions related to empathy to others.

Regarding musical training, the subnetwork of the RN network associated with musical training history includes the sensorymotor region and the inferior frontal gyrus (IFG). Sensory and motor regions are considered responsible for motion and touch processing involved in music practice, and IFG is responsible for language processing. Because the participants had experiences in single or multiple musical instruments (Supplementary Table 2), the subnetwork of the RN network associated with the number of years of musical training might reflect brain functions related to generic musical activity rather than motor skills specific to each musical instrument. Although musical activity recruits brain regions for finger and body motion, touch sensation, language, working memory, etc. (Pallesen et al.^2010^; Patel 2014; Choi et al. 2015; Pinheiro et al. 2015), the length of musical training was only associated with the sensory motor region and the IFG in the RN network. This may indicate that the RN network primarily reflects motor, procedural memory, and language related characteristics, but not the many other characteristics, of musical activity.

Although, as previous studies have shown, the role of RN is known to have evolutionarily regressed in pure motion processing, it is yet to be clarified what other crucial functions RN is responsible for. Functions of social cognition, such as empathy or music, entailing with motion or relativity may be candidates.

Clinical studies using rsfMRI have shown that connectivity changes in rsfMRI networks reflect brain disorders. Therefore, the RN network also has a potential application in studies of brain disorders. In particular, it may be worthwhile to investigate the possibility that detection of some brain disorders based on the RN network, i.e. by clarifying the association of the RN network with other cognitive functions or psychological traits in relation with brain disorders, may be more specific or sensitive than based on known brain networks.

We found that the strength of edges in the RN network was highly consistent between two relatively small sample populations as well as a larger sample population, adding further significance for the RN network. The significant association of the RN networks with IRI and the number of musical training years suggests that the network reflects cognitive functions, with the caveat that the sample was relatively small.

Taken together, we identified the intrinsic functional RN network and demonstrated that RN is involved in processing of interpersonal reactivity and musical practicing. These social or highly coordinated motor activity represent the most complex functions ever known to involve the RN, adding further evidence for the multifunctional roles of RN. These discoveries may lead to a new direction of investigations to clarify probable novel roles for RN in high-level human behavior.

## Supplementary Material

SupplementaryMethod220818_tgac037
