# Supplementary material for: Involvement of the intrinsic functional network of the red nucleus in complex behavioral processing
Source: Cereb Cortex Commun. 2022 Aug 25;3(3):tgac037. doi: 10.1093/texcom/tgac037 (PMC9491841; doi:10.1093/texcom/tgac037)
Supplement: SupplementaryMethod220818_tgac037 [file supplementarymethod220818_tgac037.docx]

Supplementary Materials

Supplementary Data

120 healthy volunteers (38 males and 82 females; age 19.0 ± 0.9) underwent an MRI study using a 3-Tesla MRI scanner (Skyra-fit; Siemens Co., Erlangen, Germany). For all subjects, rsfMRI data were acquired using the following parameters: repetition time = 1,000 ms, matrix size = 64 × 64, in-plane resolution = 3.4 × 3.4 mm2, slice thickness = 3.4 mm, and number of volumes = 480. In the rsfMRI session, subjects were asked to lie on the bed, not to wander their mind, keep their eyes open, and gently focus on the center of the visual field. The lights in the room were turned off during MRI scan.

Supplementary Tables

Supplementary Table 2

Musice experience years

and musical instruments of 21 subjects

Supplementary Table 1

Interpersonal Reactivity Index

Of 23 subjects


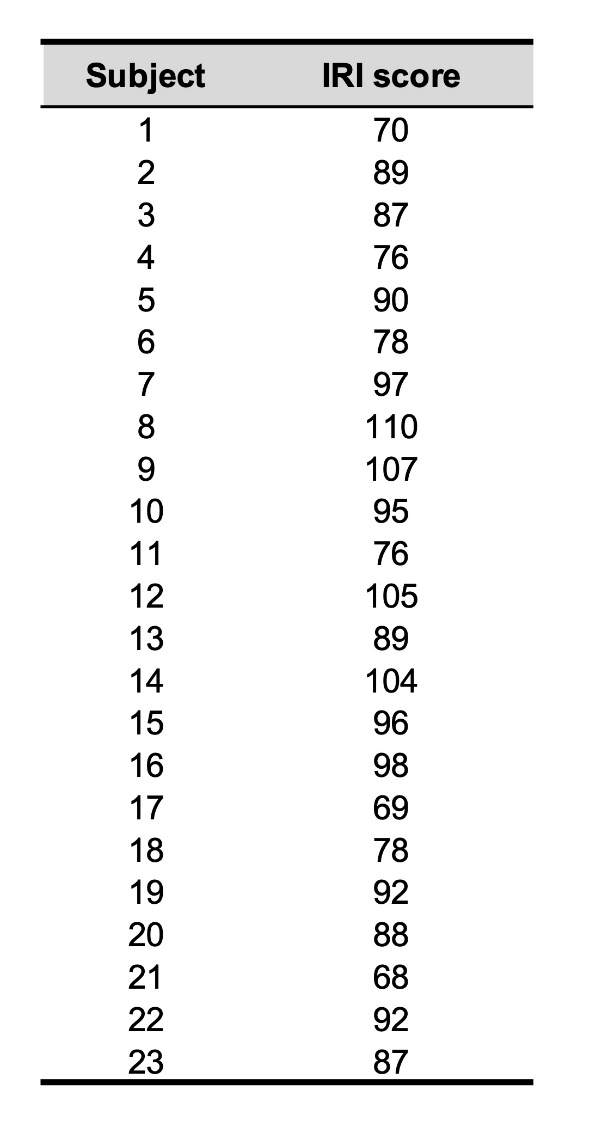


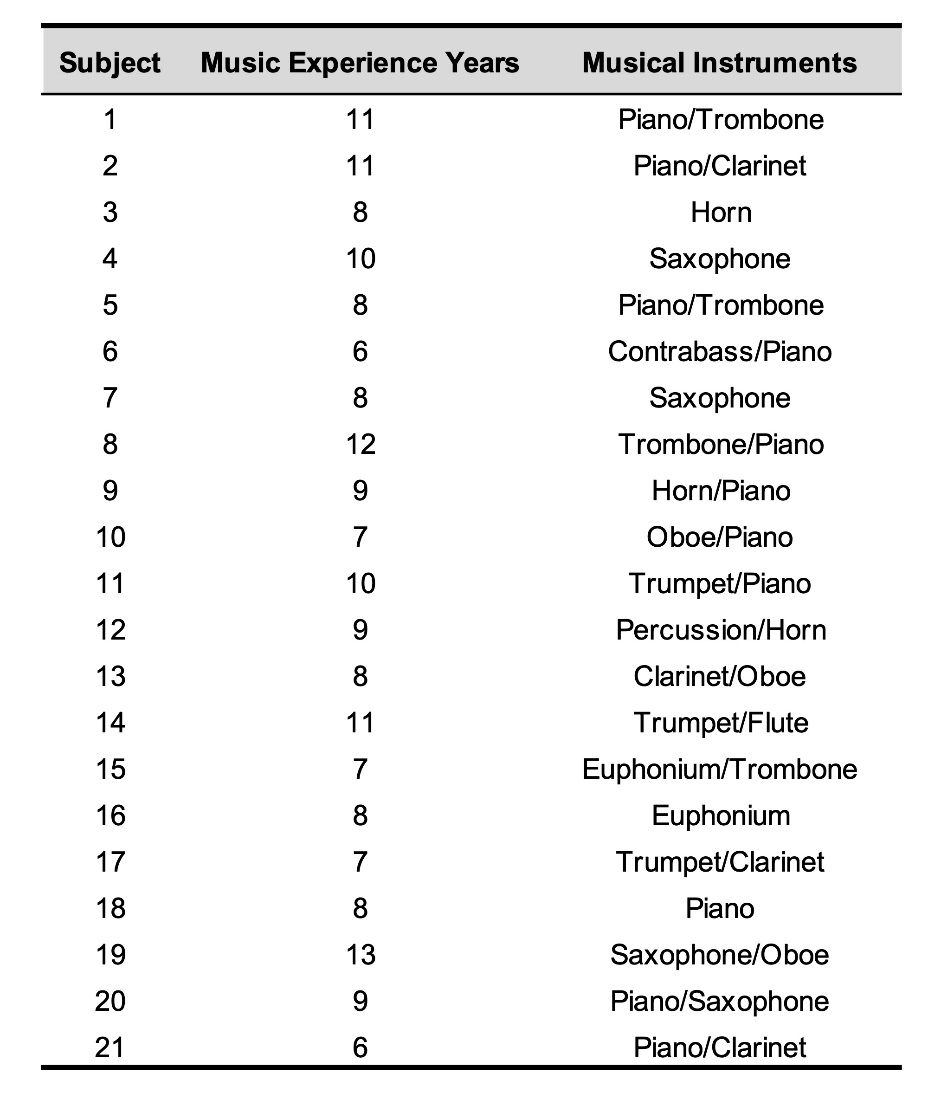


Supplementary Figures

Supplementary Figure 1


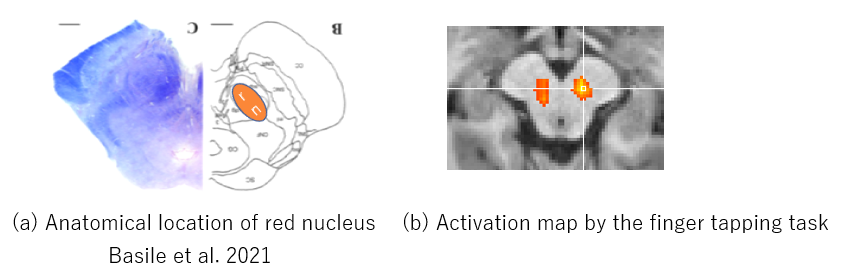


Supplementary Figures

Supplementary Figure 1


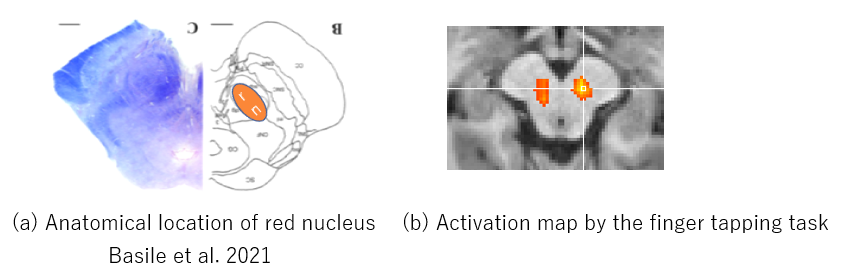


Supplementary Figure 2


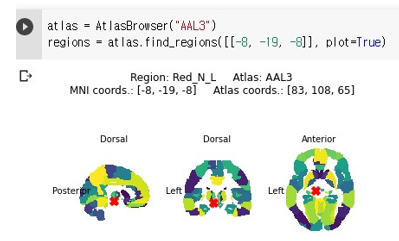


Location of MNI coordinates (-8,-19, -8) based on AAL3 atlas.
